# Sexually transmitted infections related care-seeking behavior and associated factors among reproductive age women in East Africa: a multilevel analysis of demographic and health surveys

**DOI:** 10.1186/s12889-022-14120-w

**Published:** 2022-09-09

**Authors:** Ever Siyoum Shewarega, Elsa Awoke Fentie, Desale Bihonegn Asmamaw, Wubshet Debebe Negash, Samrawit Mihret Fetene, Rediet Eristu Teklu, Fantu Mamo Aragaw, Tewodros Getaneh Alemu, Habitu Birhan Eshetu, Daniel Gashaneh Belay

**Affiliations:** 1 Department of Reproductive Health, Institute of Public Health, College of Medicine and Health Sciences, University of Gondar, P.O. Box: 196, Gondar, Ethiopia; 2 Department of Health Systems and Policy, Institute of Public Health, College of Medicine and Health Sciences, University of Gondar, Gondar, Ethiopia; 3 Department of Epidemiology and Biostatistics, Institute of Public Health, College of Medicine and Health Sciences, University of Gondar, Gondar, Ethiopia; 4 Department of Pediatrics and Child Health Nursing, School of Nursing, College of Medicine and Health Sciences, University of Gondar, Gondar, Ethiopia; 5 Department of Health Education and Behavioral Sciences, Institute of Public Health, College of Medicine and Health Sciences, University of Gondar, Gondar, Ethiopia; 6 Department of Human Anatomy, College of Medicine and Health Sciences, University of Gondar, Gondar, Ethiopia

**Keywords:** Sexually transmitted infections, Care-seeking behavior, Women, East Africa

## Abstract

**Background:**

Sexually transmitted infections are serious global public health issue, and their consequences contribute significantly to population morbidity and mortality, especially in Sub-Saharan Africa. However, there is limited information about the sexually transmitted infections related care-seeking behavior in East Africa. Therefore, this study aimed to assess the pooled prevalence of sexually transmitted infections related care-seeking behavior, and associated factors among reproductive-age women in East Africa using the recent Demographic and Health Survey.

**Methods:**

This study was based on recent Demographic and Health Survey of 8 East African countries from 2008/09 to 2018/2019. A total weighted sample of 12,004 reproductive-age women who reported sexually transmitted infections or symptoms of sexually transmitted infections in the last 12 months wereincluded. A multi-level mixed-effect logistic regression model was used and a *P*-value of < 0.05 was considered a statistically significant level for identification of individual and community level factors and AOR with a 95% l CI was computed.

**Result:**

The overall prevalence of sexually transmitted infections related care-seeking behavior among reproductive-age women in East African countries was 54.14% [95% CI: 53.25%, 55.03%]. In multilevel analysis: being age 25–34 [AOR = 1.27 95%CI: 1.15–1.41], 35–49 [AOR = 1.26 95%CI: 1.13–1.41], women who attained secondary or above education [AOR = 1.27, 95% CI: 1.09, 1.47], being in rich household [AOR = 1.27, 95% CI 1.14, 1.41], women who were currently pregnant [AOR = 1.29, 95% CI 1.13, 1.47], who had been tested for HIV [AOR = 1.99, 95% CI 1.70, 2.33], women who had one and more than one sexual partner [AOR = 1.18, 95% CI 1.05, 1.34], women who lived in urban area [AOR = 1.16, 95% CI: 1.03, 1.31] and who perceived distance from the health facility was not a big problem was [AOR = 1.13, 95% CI 1.04, 1.23] were significantly associated with sexually transmitted infections related care-seeking behavior.

**Conclusion:**

sexually transmitted infections related care-seeking behavior is relatively low as compared with other studies.. This study revealed that individual-level variables such as women's age, educational status, household wealth index, pregnancy status, ever been tested for HIV, number of sexual partners, and community-level variables such as residence and distance from a health facility were associated with sexually transmitted infections related care-seeking behavior. Therefore, public health interventions targeting uneducated women, poor households, and adolescents, as well as improving counseling and awareness creation during HIV/AIDS testing and Antenatal care visits, are vital to improving sexually transmitted infections care seeking behavior.

## Introduction

Sexually transmitted infections (STIs) are a group of clinical syndromes and infections caused by pathogens that are acquired and transmitted through sexual contact [1]. It is a serious global public health issue, and its consequences contribute significantly to population morbidity and mortality [2]. The World Health Organization (WHO) has estimated that annually approximately 374 million new cases of curable STIs such as syphilis, gonorrhea, and chlamydia, occur worldwide in 2021 [3] and sub-Saharan Africa accounts for approximately 40% of the global burden of STIs [4].

Untreated STIs could also lead to infertility, pelvic inflammatory disease (PID), ectopic pregnancy, long-term disability, severe psychological problems, cervical cancer, and pregnancy complications like premature delivery, stillbirth, low birth weight, and neonatal infections [5]. Moreover, Evidence suggested that untreated STIs can increase the risk of human immunodeficiency virus (HIV) infection and transmission fourfold [6]. These infections are substantial health and economic burden worldwide, especially in developing nations, where they account for 17% of all economic losses attributable to illness [7].

The health-seeking behavior of people with STIs, who may seek care from a variety of sources, is an essential factor for effective STI control and prevention of those complications [8]. Health Seeking Behaviors refers to individuals' efforts to identify appropriate solutions in response to illness or health concerns. In many low- and middle-income countries (LMIC) health services did not fully address women's sexual and reproductive health (SRH), including STI-related needs [9, 10].

Even though most STIs can be cured with prompt treatment, they are usually asymptomatic or go unnoticed [11]. Women are disproportionately affected; for example, gonorrhea and syphilis are asymptomatic in less than 10% of men against 50%-80% of women [12]. Because most people with STIs have mild or no symptoms, they do not seek treatment at public health institutions, while others self-medicate. Due to taboos and inhibitions around sexual and reproductive health, women with self-reported symptoms of sexual morbidity do not seek treatment [13].

Several studies showed that health care-seeking behavior is affected or influenced by different factors like lack of money, distance from the health facility, age, educational status, residence, occupation, age at first sex, number of sexual partners, use of a condom, being tested for HIV, media exposure, wealth index cultural beliefs and practices are some of the identified factors which affect the health care seeking behavior of women [13–18].

STI prevention and control have a wide range of advantages and help the achievement of Sustainable Development Goals of reducing under-five mortality, combatting infectious diseases, and providing sexual and reproductive health care [19]. The WHO established a Global Health Sector Strategy on STIs in 2016, intending to put an end to STI epidemics between 2016 and 2021 [20]. But still, the care-seeking behavior related to STIs is low.

Early detection and treatment of STIs are crucial to reducing prevalence and breaking the transmission chain of STIs [21]. In many poor and middle-income countries, sexual and reproductive health needs were not adequately met [10]. Due to sexual and reproductive health taboos and inhibitions, women with self-reported symptoms of sexual morbidity do not seek treatment [13]. The majority of previous research on the STIs related care seeking behavior in East Africa was institutional in nature, limited to particular nations, regions, or zones, and had a small sample size. However, Our study, uses nationally representative data to better understand determinants of STI related care seeking behaviora an individual and community level. Therefore, the objective of this study was to assess the pooled prevalence of STIs related care seeking behavior and associated factors among reproductive-age women in East Africa The finding of this study could help to understand women’s health-seeking practices and the underlying factors for them which can help policymakers to design policies and strategies aimed at improving the accessibility and acceptability of STI care services.

## Method and material

### Study design and setting

This study used data from the Demographic and Health Survey (DHS), which was obtained using a community-based cross-sectional study design. Since 1984, the DHS has been undertaken in over 90 countries worldwide and it is comparable to nationally representative household surveys. The DHS collects a variety of objective and self-reported information on adult fertility, reproductive health, mother and child health, mortality, nutrition, and health behaviors [22]. The benefits of DHS include high response rates, national coverage, quality interviewer training, a country-wide standardized data collection process, and long-term consistent content. [23]. As a result, the current study is based on demographic and health surveys done in East Africa over the last ten years, from 2008/09 to 2018/2019.

### Data sources, sampling technique, and study population

The data for this study were drawn from recent nationally representative DHS data conducted in 8 countries (Burundi, Ethiopia, Malawi, Kenya, Comoros, Rwanda, Uganda, and Zambia,) in East Africa over the last 10 years(2008–2018). There 20 countries in WHO regions of East Africa. In history, only 14 countries had DHS data. But, countries such as Sudan and Eritrea had no recently conducted DHS data, moreover other East African countries such as Madagascar, Mozambique, Zimbabwe, and Tanzania, had no recorded data on the STIs-related information of reproductive age women in their demographic and health survey dataset, so For this study 8 countries were included.. To ensure national representativeness, the DHS survey used a two-stage stratified sampling procedure to select survey participants [22]. In this study, we pooled the last DHS data from eight East African countries and included a weighted sample of 12,004 reproductive-age women. The survey year and total weighted sample included in this study were presented in (Table 1).

**Table 1:** The study participants by country and respective year of the survey

Country	Year of survey	Weighted frequency (n)	Percent
Burundi	2016	1,339	11.16
Ethiopia	2016	474	3.95
Kenya	2014	714	5.95
Comoros	2012	395	3.29
Malawi	2015/2016	3,183	26.52
Rwanda	2014/2015	1,429	11.91
Uganda	2016	3,857	32.13
Zambia	2018	613	5.10
Total		12,004	100

### Study variables

#### Dependent variable

Reproductive-age women (15–49 years) who had STIs or symptoms of STIs (a bad-smelling, abnormal discharge from the vaginal area or a genital sore or ulcer) in the 12 months prior to the survey and sought treatment or advice were classified as having STIs-related care-seeking behavior and coded as “Yes,” whereas those who had STIs or symptoms of STIs but did not seek treatment or advice were classified as not having STIs-related care-seeking behavior and coded as “No” [22].

#### Independent variables

We incorporated several individual/household and community-level independent variables based on available evidence on the STIs-related care-seeking behavior uptake among reproductive-age women [11, 13, 16, 17, 24–29]. The following individual/household level factors were incorporated and classified as follows: age of respondent (15–24, 25–34, and 35–49), women educational status (no education, primary and secondary and above) occupational status (working and not working), ever heard about STIs(yes and no), ever heard about HIV(yes and no), ever tested HIV(yes and no), number of the sexual partner in the last 12 months (0, one and more than one), current pregnancy status (pregnant and not pregnant), sexual debut age (≥ 15 and < 15), wealth status (poor, middle, and rich), and media exposure (media exposure consists of three variables: listening to television, listening to the radio, reading newspapers, "yes" if a woman is exposed to any of the three media sources, and no if she is not exposed to any of them).

In this study, place of residence, distance from the health facility, community level poverty, community level media exposure, and country and community level women's education were community-level factors. Distance to a health facility is categorized as ("a big problem" or "not a problem"), a big problem indicates that the distance from a woman's residence to a health facility for medical care was troublesome. If the women responded as distance was a big problem, we coded it as 0 if the women reported it as not a big problem we coded it as 1. If the women reported the distance was a big problem, we coded it as 0, and if they said it wasn't, we coded it as 1. Whereas, Individual-level factors were aggregated at the community (cluster) level to create aggregate community-level independent variables (community level poverty, community level media exposure, community level women education). After checking the distribution using the histogram, we classified them as high or low based on the distribution of proportion values ​​calculated for each community. Because the aggregate variable was not normally distributed, the median value was chosen as a classification cut-off point.

Data Management and Statistical Analysis After literature-based variable extraction, DHS data from eight East African countries were pooled. STATA version 14 was used for data extraction, recoding, and analysis. The sample was weighted to restore its representativeness, such that the overall samples represent the country's actual population. Descriptive statistics were described using frequencies, percentages, median, and interquartile ranges, and were presented using tables, figures, and narratives. After confirming the eligibility of the model, we performed a multi-level logistic regression analysis. First, a bi-variable multilevel logistic regression analysis was performed and a variable with a *p*-value < 0.20 was included in the multivariate analysis. After selecting variables for multilevel analysis, four models were fitted. Null model (no independent variables), Model I (includes only individual-level factors), Model II (community-level factors), and Model III (includes both individual-level and community-level factors). The intra-class correlation coefficient (ICC), median odds ratio (MOR), and proportional change in variance (PCV) were used to assess the random effect analysis, which is a measure of variation in treatment-seeking behaviors toward STIs across communities or clusters (PCV). The goodness of fit of the model was evaluated by deviance, and the model with the lowest deviance (Model III) was the best. Then, in the final model, a *p*-value of less than 0.05 and an Adjusted Odds Ratio (AOR) with a 95% confidence interval (CI) was used to estimate the association between individual and community-level characteristics with STI-related care-seeking behavior.

## Result

### The pooled prevalence of care-seeking behavior

**Fig. 1:**
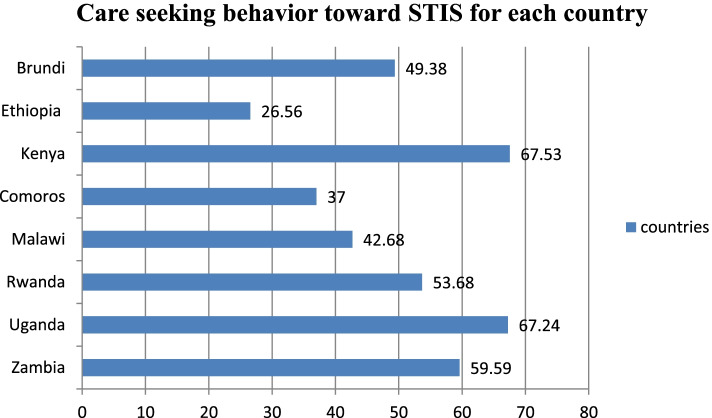
Care seeking behavior toward STIs in East Africa

The pooled prevalence of care-seeking behavior toward STIs in East African countries was 54.14% [95% CI: 53.25%, 55.03%]. The highest prevalence of care-seeking behavior toward STIs was found in Kenya (67.53%), while the lowest prevalence was found in Ethiopia (26.56%) (Fig. 1).

### Socio-demographic and economic characteristics of the respondents

A total of 12,004 (weighted) reproductive age women who reported STIs or symptoms of STIs were included in this study. The median age of the participants were 30 (IQR: 24–36) years with 4,747(39.54%) women aged between 25 and 34 years. Over half (58.03%) of respondents had attained primary education and the majority of the respondents (80.31%) were currently working. Moreover, more than three fourth (89.72%) of the respondent had media exposure (Table 2).

**Table 2:** Socio-demographic and economic characteristics of reproductive age women (15-49yrs) reported STIs or STIs symptoms in East Africa

Variables	Weighted frequency (n)	Percentage
**Age of the respondents**		
15–24	3,435	28.62
25–34	4,747	39.54
35–49	3,822	31.84
**Educational status of the respondent**		
No formal education	2,057	17.41
Primary education	6,966	58.03
Secondary education and above	2,981	24.83
**Occupation of the respondent**		
Working	9,641	80.31
Not working	2,363	19.69
**Current marital status**		
Unmarried	1,180	9.83
Married	8,965	74.68
Formerly married	1,859	15.49
**Wealth Index**		
Poor	4,549	37.90
Middle	2,399	19.98
Rich	5,056	42.12
**Media exposure**		
Yes	10,770	89.72
No	1,234	10.28

### Reproductive health characteristics of the respondents

The majority of the respondents (99.21%) ever heard about STIs and about 89.35% of respondents had been tested for HIV. Of the total respondents, 64.12% of respondents were sexually active in the last 4 weeks and the majority of the respondents (85.01%) had one sexual partner in the last 12 months (Table 3).

**Table 3:** Reproductive health characteristics of reproductive age women (15-49yrs) reported STIs or STIs symptoms in East Africa

Variables	Weighted Frequency (n)	Percentage
**Age at first sex**		
≥ 15	10,009	83.38
< 15	1,995	16.62
**Ever heard about STIs**		
Yes	11,909	99.21
No	95	0.79
**Ever heard about AIDS**		
Yes	11,852	98.73
No	152	1.27
**Ever been tested for HIV**		
Yes	10,725	89.35
No	1,279	10.65
**Current pregnancy status**		
Pregnant	1,315	10.95
Not pregnant	10,689	89.05
**Recent sexual activity**		
Not active in last 4 weeks	3,947	32.88
Active in last 4 weeks	8,057	64.12
**Number of sexual partner in the last 12 months**		
0	1,366	11.38
1	10,209	85.05
More than one	429	3.58

### Community level variables

More than three fourth (77.11%) of respondents were from rural communities. More than half (56.8%) of the respondents were from countries where distance from the health facility was not a big problem. Half (50.08%) of them were from countries with high uneducated levels and 47.6% of respondents were from countries with high poverty levels (Table 4).

**Table 4:** Community level variables in East Africa

Variables	Weighted frequency (n)	Percentage
**Residence**		
Urban	2,747	22.89
Rural	9,257	77.11
**Community-level media exposure**		
Low	6,132	51.08
High	5,872	48.92
**Community-level poverty**		
Low	6,290	52.4
High	5,714	47.6
**Community-level illiteracy**		
Low	5,993	49.92
High	6,011	50.08
**Distance from the health facility**		
Not big problem	6,819	56.80
Big problem	5,185	43.20

### Random effects and model fitness

The fixed effects (a measure of association) and the random intercepts for care-seeking behavior toward STIs are presented in Table 5. The results of the null model revealed that variance [country variance = 0.271; standard error (SE) = 0.31], indicating the existence of statistically significant differences between countries care seeking behavior toward STIs among women reported STIs or symptoms of STIs. This was further supported by the ICC in the null model which showed that about 7.62% of the variation of care-seeking behavior toward STIs among women who reported STIs or symptoms of STIs was attributed to the difference in country-level factors. Moreover, the MOR was 1.64 [95%CI: 1.55,1.74] which implied that the odds of care-seeking behavior toward STIs were 1.64 times higher when the respondents move from low to high-risk communities. This showed the existence of significant heterogeneity in care-seeking behavior toward STIs across different countries.

**Table 5:** Random effect and model comparison

Parameters	Null model	Model I	Model II	Model III
Community Variance(SE)	0.271(0.31)	0.241(0.30)	0.210(0.28)	0.200(0.28)
ICC	7.62	6.83	6.01	5.75
PCV	Ref	11.1	22.4	25.9
MOR	1.64	1.59	1.56	1.53
Log likelihood	-8158.95	-7922.12	-7809.39	-7698.46
Deviance	16,317.9	15,844.24	15,618.78	15,396.92

Besides, the final model(model III) PCV indicates that about 25.9% of the variation of care-seeking behavior toward STIs among women who reported STIs or symptoms of STIs was attributable to both individual-level and community-level factors. Regarding model comparison, we used deviance to assess model fitness. Consequently, the model with the lowest deviance value (Model III) was found to be the best-fitted model (Table 5).

### Factors associated with care-seeking behavior toward STIs in East Africa

In the final model (model III), where both the individual and community level factors were fitted simultaneously; age of the respondent, educational status, household wealth index, ever been tested for HIV, age of the respondent, number of sexual partners, current pregnant status, from individual-level factors and residence, country and distance from health facility from the aggregate community level factors were significantly associated with care seeking behavior toward STIs.

The odds of STIs-related care-seeking behavior of women whose ages were 25–34 and 35–49 were 1.27 times [AOR = 1.27; 95%CI: 1.15–1.41] and 1.26 times [AOR = 1.26 95%CI: 1.13–1.41] higher as compared to women who were aged 15–24 years respectively. Women who attained secondary or above education were 1.27 times [AOR = 1.27, 95% CI: 1.09, 1.47] higher odds of and STIs-related care-seeking behavior compared to women who did not have formal education.

The odds of STIs-related care-seeking behavior of women from a household with rich wealth status was 1.27 times [AOR = 1.27, 95% CI 1.14, 1.41] higher than women from a poor household. The odds of STIs related care-seeking behavior of the women who had been tested for HIV were 1.99 times [AOR = 1.99, 95% CI 1.70, 2.33] higher than their counterparts.

The odds of STIs-related care-seeking behavior of women who were currently pregnant was 1.29 times [AOR = 1.29, 95% CI 1.13, 1.47] higher than non-pregnant women. The odds of STIs related care-seeking behaviors were 1.18 [AOR = 1.18, 95% CI 1.05, 1.34] and 1.27 [AOR = 1.27, 95% CI 1.00, 1.62] times higher among women who had one and more than one sexual partner in the last 12 months compared to women who had no a sexual partner in the last 12 months.

The odds of STIs related care-seeking behavior among women in Burundi, Ethiopia, Kenya, Comoros, Malawi, Rwanda and Zambia were decreased by 49% [AOR = 0.51, 95% CI: 0.41, 0.63], 78% [AOR = 0.22, 95% CI: 0.17, 0.30], 55% [AOR = 0.45, 95% CI: 0.33, 0.62], 60% [AOR = 0.40, 95% CI: 0.33, 0.49], 45% [AOR = 0.55, 95% CI: 0.45, 0.68] and 26% [AOR = 0.74, 95% CI: 0.58, 0.95] compared to women in Kenya, respectively. Women who lived in urban area was 1.16 times [AOR = 1.16, 95% CI: 1.03, 1.31] higher odds of STIs related care-seeking behavior than rural women. The odds of STIs related care-seeking behavior of women who perceived distance from the health facility was not a big problem was 1.13 times [AOR = 1.13, 95% CI 1.04, 1.23] higher than women who perceived distance from health facility was a big problem (Table 6).

**Table 6:** determinant of care seeking behavior towards STIs among reproductive age women in East Africa

Variables	Care seeking behavior toward STI		Model I AOR (95% CI)	Model II AOR (95%CI)	Model III AOR (95%CI)
	**No**	**Yes**			
**Age**					
15–24	1,674	1,761	1		1
25–34	2,056	2,691	1.25(1.13–1.37)		1.27(1.15–1.41)*
35–49	1,775	2,047	1.25(1.12–1.39)		1.26(1.13–1.41)*
**Educational status**					
No formal education	1,131	926	1		1
Primary education	3,262	3,704	1.22 (1.09–1.36)		1.03(0.91–1.16)
Secondary education and above	1,111	1,870	1.67(1.45–1.92)		1.27(1.09–1.47)*
**Occupation of the respondent**					
Working	4,314	5,327	1.15(1.04–1.27)		1.07(0.96–1.19)
Not working	1,190	1,173	1		1
**Wealth index**					
Poor	2,327	2,221	1		1
Middle	1,144	1,255	1.05 (0.95–1.17)		1.06(0.95–1.18)
Rich	2,032	3,023	1.27 (1.15–1.39)		1.27(1.14–1.41)*
**Media exposure**					
Yes	5,068	5,702	0.82(0.71–0.95)		0.90(0.77–1.04)
No	437	797	1		1
**Age at first sex**					
≥ 15	4,524	5,485	1.03(0.93–1.15)		1.08(0.97–1.21)
< 15	981	1,014	1		1
**Ever heard about STIs**					
Yes	5,430	6,479	1.63(0.76–3.49)		1.26(0.58–2.71)
No	75	20	1		1
**Ever heard about AIDS**					
Yes	5,394	6,458	0.88(0.49–1.59)		0.89(0.49–1.61)
No	111	41	1		1
**Ever been tested for HIV**					
Yes	4,639	6,086	2.40(2.08–2.77)		1.99(1.70–2.33)*
No	866	413	1		1
**Current pregnancy status**					
Pregnant	520	795	1.35(1.19–1.53)		1.29(1.13–1.47)*
Not pregnant	4,984	5,705	1		1
**Number of sexual partner in the last 12 months**					
0	711	655	1		1
One	4,619	5,590	1.25(1.10–1.41)		1.18(1.05–1.34)*
More than one	174	255	1.48(1.17–1.88)		1.27(1.00–1.62)*
**Country**					
Burundi	678	661		0.50(0.41–0.62)	0.51(0.41–0.63)*
Ethiopia	348	126		0.17(0.13–0.23)	0.22(0.17–0.30)*
Kenya	232	482		1	1
Comoros	249	146		0.28(0.21–0.37)	0.45(0.33–0.62)*
Malawi	1,825	1,358		0.39(0.33–0.48)	0.40(0.33–0.49)*
Rwanda	662	767		0.57(0.46–0.70)	0.55(0.45–0.68)*
Uganda	1,263	2,594		1.04(0.86–1.25)	1.02(0.84–1.23)
Zambia	248	365		0.72(0.57–0.93)	0.74(0.58–0.95)*
**Residence**					
Urban	1,022	1,725		1.40(1.26–1.56)	1.16(1.03–1.31)*
Rural	4,483	4,774		1	1
**Community level media exposure**					
Low	2,718	3,414		1	1
High	2,786	3,086		0.99(0.89–1.10)	1.02(0.91–1.14)
**Community level poverty**					
Low	2,739	3,551		1.02(0.92–1.14)	0.97(0.86–1.08)
High	2,766	2,948		1	1
**Community level illiteracy**					
Low	2,570	3,423		1.09(0.98–1.22)	1.07(0.96–1.19)
High	2,935	3,076		1	1
**Distance from the health facility**					
Not big problem	2,898	3,921		1.17(1.08–1.27)	1.13(1.04–1.23)*
Big problem	2,607	2,578		1	1

*AIDS* Acquired Immunodeficiency Syndrome, *STIs* Sexually Transmitted Infections, *AOR* Adjusted Odds Ratio, *CI* Confidence Interval, * = *p* value < 0.05

## Discussion

This study aimed to assess the pooled prevalence and associated factors of STIs-related care-seeking behavior in east Africa using the pooled DHS data. The pooled prevalence of STIs related care-seeking behavior in East Africa in this study was 54.14% (95% CI: 53.2%, 55.0%), ranging from 26.56% in Ethiopiato 67.53% in Kenya. The finding was much higher than studies conducted,Ghana Accra 35% [17], Nigeria 48% [24], India 14% [13] Bangladesh 50% [25]. However, this finding is lower than the studies done in Iran 68.85% [11] and Dehradun India 63% [27]. The discrepancy might be due to the difference in socioeconomic status, cultural norms, access to media, information, knowledge, and access and availability to health facilities across different countries [16].

In this study after adjusting for individual and community level factors, we found age of women, educational status, household wealth index, being tested for HIV/AIDS, current pregnancy status, and the number of the sexual partner from individual-level factors whereas residence and distance from health facility from community level factors were significantly associated with STIs related care-seeking behavior.

This study shows that women who were aged 25–34 and 35–49 years were more likely to have STIs-related care-seeking behavior as compared to women who were aged 15–24. This finding is supported by the other studies conducted in Nigeria [24], Pakistan [30], and Iran [27]. The possible reason might be because older women are more aware of the reproductive health care available at health facilities than younger women [24]. The other possible explanation might be most young women are embarrassed and ashamed to go to the clinic for treatment since it is a sexual related issue [10].

The findings of the study show that women who attained secondary education and above were more likely to have STIs-related care-seeking behavior compared to women who did not have formal education. This finding is consistent with studies conducted in India [31], Tamilnadu, India [13]. The explanation for this finding could be that education is the foundation for many things, and thus educated people have greater access to information and can apply health education messages they receive from health institutions [32]. Furthermore, education plays an important role in boosting women's confidence and ability to make decisions regarding their health [16].

The odds of STIs-related care-seeking behavior of women from a household with rich wealth status was higher than women from a poor household. This finding is supported by studies done Ghana, India [31], Nigeria [24], India [13]. The reason might be wealth is a crucial indication of access to most health services, as wealthy individuals are more likely to pay for their services and women with good economic status are more likely to be able to overcome financial barriers to access health care services [16, 32]. Additionally, wealthy people might be more likely to access information through media like radio and television, and they might not be concerned about healthcare costs [33].This study evidenced that the odds of STIs-related care-seeking behavior among women who had ever been tested for HIV/AIDS (Human Immunodeficiency Virus/ Acquired Immunodeficiency Syndrome) were higher as compared to women who had not ever been tested for HIV/AIDS. The possible explanation might be women who had ever been tested for HIV/AIDS get better counseling and awareness about STIs and treatment during their visit. This implies that the health sector should strengthen counseling and awareness creation during testing for HIV/AIDS to increase care-seeking behavior toward STIs [34].

This study showed that the odds of STIs-related care-seeking behavior of women who were currently pregnant was higher than non-pregnant women. This finding is consistent with a study done in Ethiopia [16]. The possible explanation might be pregnant women receive STI counseling and education during their antenatal care (ANC) visit. The other possible explanation might be WHO recommended pregnant women should be screened for STIs during their ANC visit [16].

This study showed that the odds of STIs-related care-seeking behavior were higher among women who had one and more than one sexual partner in the last 12 months compared to women who had no a sexual partner in the last 12 months. The possible explanation might be women who start having sex and have multiple sexual partners suspect themselves that they might have STIs so they are more likely to seek care [34].

This study also revealed that residency is associated with STIs related care-seeking behavior. Women who lived in urban areas had higher odds of STIs-related care-seeking behavior than rural women. This finding is similar to a study done in India [31]. This might be due to women who live in urban had better access to services and since they are highly exposed to media they had access to information [35]. As a result, women who have from rural area may become less motivated to seek care compared with their counterparts. Besides, women residing in rural areas have limited access for education and low chance of getting health information than women residing in urban [36]. This strong association implied that it is crucial to educate rural women about STI infections, early treatment as well as building facilities that are easily accessible to them.This study evidenced that there is an association between distance from health facilities and STIs-related care-seeking behavior. The odds of STIs-related care-seeking behavior of women who perceived distance from the health facility was not a big problem higher than women who perceived distance from health facility was a big problem. This finding is consistent with a study done in India [13]. The possible explanation might be women who perceived distance from health facilities as not a big problem do not face the additional cost of transport and time which is attributed to distance so they are more likely to seek care [37]. These findings imply interventions that aim to improve women’s STIs-related care-seeking behavior should focus on low socioeconomic rural women living far from health facilities.

### Strength and limitations of the study

The weighted nationally representative data from eight East African countries with a large sample size were used in this study. In order to provide credible standard error and estimate, multilevel analysis was employed to accommodate the hierarchical nature of the DHS data. Furthermore, because it is based on national survey data, the study has the potential to provide information to policymakers and program planners to build appropriate national and regional interventions. However, this study had a flaw in that the DHS survey was based on the respondents' reports, which could lead to recall bias. Due to the cross-sectional character of the study, we are unable to prove a cause-and-effect link between STI-related care-seeking behavior and independent variables. Furthermore, the variance in DHS study periods may not reflect the real picture of STI-related care-seeking behavior in the region.

### Implication to research and policy

The aim of this study was to assess the pooled prevalence of STIs related care seeking behavior and associated factors among reproductive-age women in East Africa. The finding of this study could help to understand women’s health-seeking practices and the underlying factors for them which can help policymakers to design policies and strategies. This study shows that youth women, uneducated women, women from rural areas, women from poor households and women who perceived distance from health facilities as a big problem had poor STIs-related care-seeking behavior as compared to their counter parts. This could have implied that there is a need for an intervention for disadvantaged women for effective STI control and prevention. This study also showed that testing for HIV/AIDS and having ANC visit increase the health seeking behavior of women towards STI. This association implies that policy makers should design strategies that strengthen counseling and awareness creation during HIV/AIDS testing and ANC visits to increase care-seeking behavior toward STIs.

## Conclusion and recommendations

This study showed that the STIs related care-seeking behavior remains a major public health problem in East Africa with significant variation across countries. Individual level variables such as age, educational status, wealth index, ever tested for HIV, being pregnant, number of sexual partners, and community level variables such as residence, distance from the health facility, and country were significant predictors of STIs-related care-seeking.

behavior. Therefore, public health interventions targeting uneducated women, poor households, and adolescents, as well as improving counseling and awareness creation during HIV/AIDS testing and ANC visits, are critical in raising their understanding of the necessity of STIs care-seeking behavior. Furthermore, Strategies and policies should be designed to increase the accessibility of healthcare services, and financial support that allows women from poor households to use health services will be beneficial.

## Data Availability

Data for this study were sourced from Demographic and Health surveys (DHS) and are available here: http://dhsprogram.com/data/available-datasets.cfm.

## Abbreviations

AIDS: Acquired Immunodeficiency Syndrome
ANC: Antenatal Care
AOR: Adjusted Odds Ratio
CI: Confidence Intervals
COR: Crude Odds Ratio
DHS: Demographic and Health Survey
EAs: Enumeration Areas
HIV: Human Immunodeficiency Virus
ICC: Intra-Cluster Correlation
OR: Odds Ratio
PCV: Proportional Change in Variance
STIs: Sexually Transmitted Infections
WHO: World Health Organization
